# Exploring the relationship between HCMV serostatus and outcomes in COVID-19 sepsis

**DOI:** 10.3389/fimmu.2024.1386586

**Published:** 2024-05-08

**Authors:** Dominik Ziehe, Alexander Wolf, Tim Rahmel, Hartmuth Nowak, Helge Haberl, Lars Bergmann, Katharina Rump, Birte Dyck, Lars Palmowski, Britta Marko, Andrea Witowski, Katrin Maria Willemsen, Stephanie Pfaender, Martin Eisenacher, Moritz Anft, Nina Babel, Thilo Bracht, Barbara Sitek, Malte Bayer, Alexander Zarbock, Thilo von Groote, Christian Putensen, Stefan Felix Ehrentraut, Christina Weisheit, Michael Adamzik, Matthias Unterberg, Björn Koos

**Affiliations:** ^1^ Klinik für Anästhesiologie, Intensivmedizin und Schmerztherapie, Universitätsklinikum Knappschaftskrankenhaus Bochum, Bochum, Germany; ^2^ Department of Molecular and Medical Virology, Ruhr University Bochum, Bochum, Germany; ^3^ Medizinisches Proteom-Center, Ruhr-University Bochum, Bochum, Germany; ^4^ Medical Proteome Analysis, Center for Proteindiagnostics (PRODI), Ruhr University Bochum, Bochum, Germany; ^5^ Center for Translational Medicine, Medical Clinic I, Marien Hospital Herne, University Hospital of the Ruhr-University Bochum, Herne, Germany; ^6^ Klinik für Anästhesiologie, Operative Intensivmedizin und Schmerztherapie, Universitätsklinikum Münster, Münster, Germany; ^7^ Klinik für Anästhesiologie und Operative Intensivmedizin, Universitätsklinikum Bonn, Bonn, Germany

**Keywords:** viral sepsis, COVID-19 risk stratification, human cytomegalovirus, cross-reactive CD8^+^ T-cells, COVID-19 survival

## Abstract

**Background:**

Sepsis, a life-threatening condition caused by the dysregulated host response to infection, is a major global health concern. Understanding the impact of viral or bacterial pathogens in sepsis is crucial for improving patient outcomes. This study aimed to investigate the human cytomegalovirus (HCMV) seropositivity as a risk factor for development of sepsis in patients with COVID-19.

**Methods:**

A multicenter observational study enrolled 95 intensive care patients with COVID-19-induced sepsis and 80 post-surgery individuals as controls. HCMV serostatus was determined using an ELISA test. Comprehensive clinical data, including demographics, comorbidities, and 30-day mortality, were collected. Statistical analyses evaluated the association between HCMV seropositivity and COVID-19 induced sepsis.

**Results:**

The prevalence of HCMV seropositivity did not significantly differ between COVID-19-induced sepsis patients (78%) and controls (71%, p = 0.382) in the entire cohort. However, among patients aged ≤60 years, HCMV seropositivity was significantly higher in COVID-19 sepsis patients compared to controls (86% vs 61%, respectively; p = 0.030). Nevertheless, HCMV serostatus did not affect 30-day survival.

**Discussion:**

These findings confirm the association between HCMV seropositivity and COVID-19 sepsis in non-geriatric patients. However, the lack of an independent effect on 30-day survival can be explained by the cross-reactivity of HCMV specific CD8^+^ T-cells towards SARS-CoV-2 peptides, which might confer some protection to HCMV seropositive patients. The inclusion of a post-surgery control group strengthens the generalizability of the findings. Further research is needed to elucidate the underlying mechanisms of this association, explore different patient populations, and identify interventions for optimizing patient management.

**Conclusion:**

This study validates the association between HCMV seropositivity and severe COVID-19-induced sepsis in non-geriatric patients, contributing to the growing body of evidence on viral pathogens in sepsis. Although HCMV serostatus did not independently influence 30-day survival, future investigations should focus on unraveling the intricate interplay between HCMV, immune responses, and COVID-19. These insights will aid in risk stratification and the development of targeted interventions for viral sepsis.

## Introduction

Sepsis is an acute, life-threatening syndrome with millions of incidences each year (1). Up to 2020 the main pathogen inducing sepsis were bacteria (2, 3). However, similarly to bacterial pathogens, viruses are also capable of inducing critical conditions resulting in organ dysfunction and an increased Sequential Organ Failure Assessment (SOFA) Score (4) fulfilling the recent definition of sepsis [Sepsis-3 (5)]. During the COVID-19 pandemic up to 80% of sepsis cases were virally induced (6). In the post-pandemic era, the rate of virally sepsis has decreased; however, COVID-19 sepsis remains prevalent in ICUs worldwide, and it has guided the interest of clinicians towards virally induced sepsis, that was most likely underdiagnosed before (7).

In response to invasive bacterial pathogens, the human immune reaction initially involves the activation of the innate immunity. This consists of the complement-system as a non-cellular component as well as macrophages and neutrophile granulocytes and others as a cellular component, which opsonize, engulf and destroy bacteria. This is followed by the activation of a slower but more specific adaptive immune response. During the adaptive immune response, B-cells start the production of highly specific antibodies. Furthermore, T-cells that specifically target the invading bacteria are expanded.

In contrast, viral pathogens are intracellular and can partly evade from detection by the immune system. Thus, the immune response to viral infections involves the production of cytokines and specific interferons leading to

1. auto- and paracrine cellular effects, inhibiting intracellular viral replication (8) and
2. an activation of cytotoxic T-cells (CD8^+^) to eliminate infected cells.

Therefore, a virus-specific cascade following an interferon-driven network with type 1 interferon (e.g. IFN-α) leading towards an inhibition of viral replication (9) is described. An inhibition of IL-10 and an upregulation of IL-16 (10) is observed but still incompletely understood and most probably not homogenous in different viral entities. During the pre-pandemic era, influenza was the predominant pathogen, associated to viral sepsis (2, 3).

The virus’ ability to evade the host’s immune defense and cause continuous inflammatory damage is accompanied by high levels of TNF-α and IL-6 as well as reduced IFN-γ expression (7). However, different viruses will most likely show different approaches to evading the immune defense (7).

In SARS-CoV-2-sepsis, the initially described cytokine storm turned out to be less pronounced, with moderate levels of IL-6 (11, 12) compared to bacterial sepsis.

Furthermore, an imbalance and predominance of non-type-1 cytokines (such as IL-4, or IL-17) is capable to draw the immune system towards an inappropriate response, leading to exhaustion of T-cells with ineffective clearance of viral-infected cells (7).

The immune response in COVID-19 sepsis is believed to differ significantly from bacterial immunity, characterized by a delayed and less severe response, as indicated by IL-6 serum concentrations (11, 12). When compared to Influenza, COVID-19-sepsis also develops with delayed symptoms and a prolonged inflammatory phase (12). The intricate immunological landscape involves a nuanced interplay of cytokines and key immunomodulatory elements, including Type 1 and Type 2 interferons, thereby demarcating a distinct divergence between bacterial and viral immune responses (12). Specifically, the interferon response in severe COVID-19 is released later and less pronounced than in Influenza-induced sepsis, leading to longer disease duration (12). In this multifaceted milieu, various host-related factors exert influence on immune defenses, encompassing age, genetic predisposition (13) and comorbidities such as pulmonary diseases (14), cardiovascular diseases (15, 16) and obesity (17). Notably, the identification of human cytomegalovirus (HCMV) serostatus as an independent predictor of survival in bacterial sepsis has added a significant layer to our understanding of sepsis risk factors (18).

HCMV is a herpes virus that, subsequent to the primary active infection, remains in the host’s body in a latent form detected by seropositivity. The virus re-activates frequently during life priming the immune system towards the anti-viral response. Reactivation of HCMV is mainly recognized during impaired immunity like transplant-related immunosuppression or during severe infection, often focused on sepsis. Here, reactivation is considered as a worsening factor regarding mortality, ICU-and hospital-duration and other secondary complication (19–23). Clear evidence for antiviral prophylaxis in these circumstances is still missing (24). A potential worsening effect of HCMV-reactivation on the clinical course of patients is also described in severe SARS-CoV-2, but this did not impact on patient’s mortality (25) except in the very elderly (26). As a mechanistic link between HCMV-reactivation and a propagated SARS-CoV-2 infection, an upregulation of the ACE2-receptor in lung epithelial cells driven by HCMV is discussed (27). Furthermore, a study by Choi et al. (28) describes CD8^+^ T-cell exhaustion following HCMV-reactivation.

Apart from HCMV-reactivation, HCMV in a controlled stage (latency) continuously concerns the human immunity and, in fact, HCMV seropositive patients have been reported to frequently have up to 20% of CD8^+^ T-cells specific for HCMV (29–31) a number that only increases with age.

The narrative, however, extends further. In the context of COVID-19, HCMV emerges as a consequential player, associated with heightened hospitalization (32) and ICU admission, particularly in individuals under the age of 60 (33). Despite the relatively modest cohort size in which the association regarding ICU-admission was observed, the findings suggest a complex interplay between SARS-CoV-2 and HCMV.

Moreover, HCMV has the ability to disrupt the antigen presentation process of T- and NK-cells and affect the surface maintenance of TLR4 and TLR5 on HCMV-infected cells, consequently altering immune system cascades (34). Therefore, HCMV-induced impairment of the immune system may have a significant impact on the host’s immunity during a subsequent COVID-19 infection and contribute to the development of secondary infections. Additionally, latent HCMV infection may alter the host’s response to SARS-CoV-2 vaccination, as has been observed with other viral vaccinations such as influenza (35).

In view of these intriguing observations, our study endeavors to elucidate the impact of HCMV serostatus on 30-day survival and immune response in COVID-19 sepsis, drawing parallels with the established association between HCMV and bacterial sepsis.

## Materials and methods

### Patient recruitment and study design

This multicenter study was registered at the DRKS (DRKS00026184) and approved by the local ethics board of the Medical Faculty of Ruhr-University Bochum (Protocol No. 18-6606-BR) and the corresponding ethics boards of each study site. As part of the CovidDataNet.NRW project, we enrolled 95 intensive care patients with COVID-19-induced sepsis (severe COVID-19) from three different centers when SEPSIS-3 criteria were met. The recruitment period was from August 1, 2021, to March 31, 2022, and clinical data were collected in an observational approach. To be included in this study, patients had to meet the following criteria: evidence of infection with SARS-CoV-2 and evidence of underlying sepsis with an increased SOFA score of at least two points. Additionally, patients had to be aged 18 or above and provide informed consent. Beyond that, we selected 80 patients who had undergone abdominal surgery as a control group. Blood samples have been collected within 36 h after sepsis diagnosis at the University hospital Knappschaftskrankenhaus Bochum (KKB), University hospital Münster (UKM), and University hospital Bonn (UKB).

### Determination of CMV serostatus (IgG) via ELISA

The SERION ELISA classic Cytomegalovirus IgG Kit (Institut Virion\Serion GmbH, Würzburg, Germany) was used to determine the IgG concentration in the patients’ blood sera. According to the manufacturer’s instructions, 100 µL of diluted samples (1:100) and respective controls were added to microtiter test wells and incubated for 60 minutes at 37°C in a wet chamber. After four washing steps, 100 µL of IgG conjugate solution was added and incubated for 30 minutes at 37°C. Four more washing steps were conducted, followed by the addition of 100 µL substrate solution. After 30 minutes at 37°C, the reactions were stopped by adding 100 µL stopping solution to each well. The optical densities (OD) were determined using a microplate reader (CLARIOstarPLUS, BMG LABTECH, Germany). OD values were measured at a wavelength of 405 nm and analyzed by CLARIOstar Data Analysis software. The values were normalized to a standard curve, and units were calculated. Samples were classified as CMV-IgG positive when 35 or more units were detected.

### PBMC isolation

PBMCs of COVID-19 patients were isolated by subjecting the obtained blood samples to Ficoll density gradient centrifugation (GE Healthcare Europe, Freiburg, Germany). Subsequently, the phase containing the PBMCs was collected. Following erythrocyte lysis, the collected PBMCs were stored at -196°C until use.

### Immunophenotyping

Upon thawing, PBMCs were stained with 25 µl master mix, containing the optimal concentrations of each antibody, for 10 min at room temperature in the dark. Erythrocytes were lyzed using RBC Lysis Buffer (BioLegend, San Diego) for 10 min at room temperature in the dark and samples were immediately acquired on a CytoFlex flow cytometer (Beckman Coulter, Brea). Quality control was performed daily using the recommended CytoFlex Daily QC Fluorospheres (Beckman Coulter, Brea). No modification to the compensation matrix was required throughout the study.

### Clinical data

Medical data, including laboratory values, vitals, demographics, point-of-care diagnostics, and length of ICU stay, were stored in a comprehensive database (CentraXX software, Kairos GmbH, Bochum, Germany) and pseudonymized according to the obligations of the Ethics Committee.

### Statistics

Statistical analyses were performed using SPSS software Version 28 (IBM, Canada). Categorical variables were evaluated using Fisher’s exact test, while continuous variables were first subjected to a Kolmogorov-Smirnov test to assess normality. If variables were normally distributed, they were evaluated using Student’s t-test for independent samples. If variables were not normally distributed, they were subjected to a Mann-Whitney U test. Kaplan-Meier curves with subsequent log-rank tests were generated to depict 30-day survival as a function of CMV serostatus in COVID-19 patients.

## Results

### Study design

**Figure 1:**
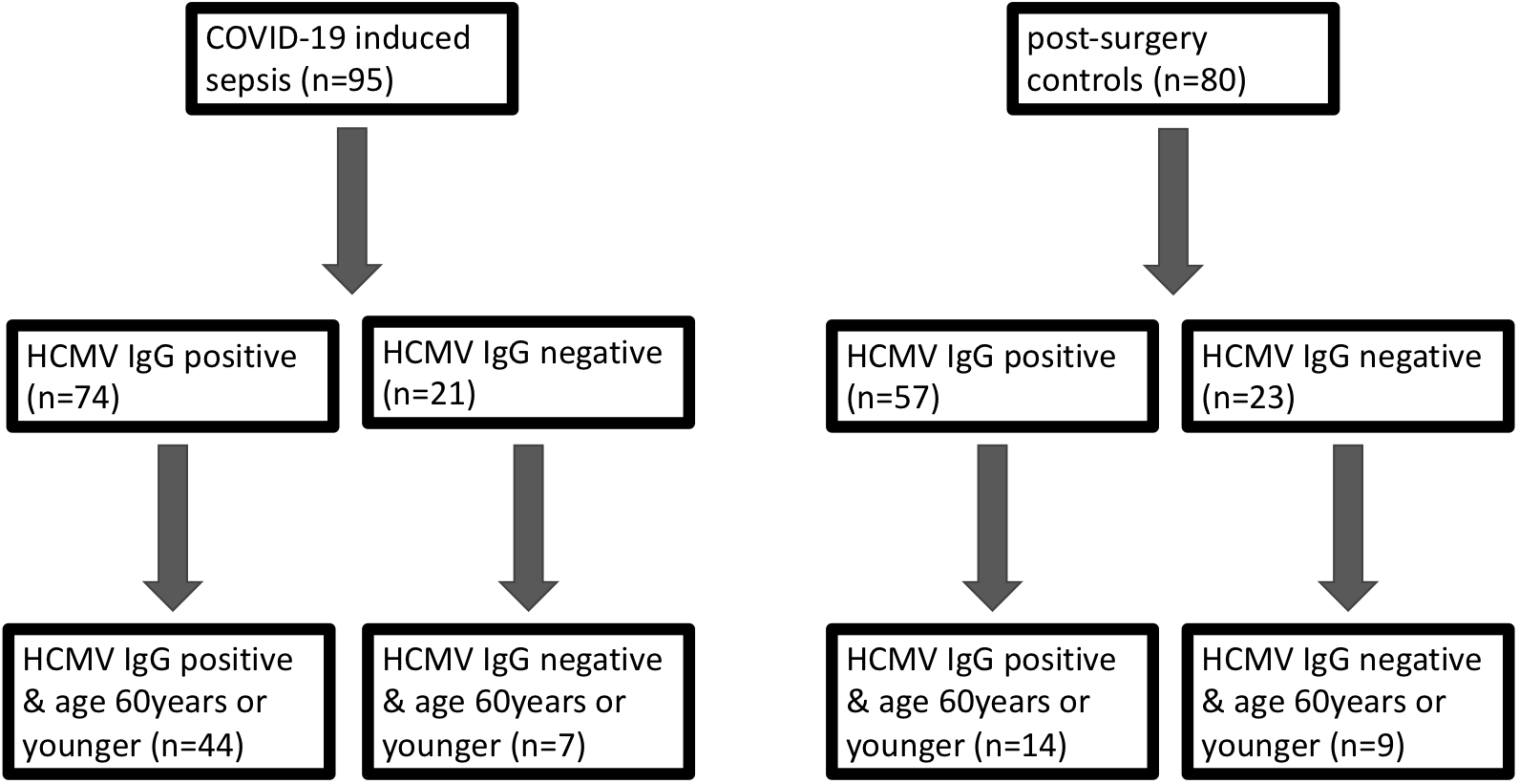
Grouping of patients with COVID-19 induced sepsis (left) and control subjects without infection (right) according to their CMV serostatus on day 1 and subsequently according to age (<= vs >60 years).

We systematically assessed the impact of HCMV serostatus on the 30-day mortality in the entire cohort of COVID-19 patients, comparing them to pre-pandemic post-surgery individuals. An additional focus was directed towards the non-geriatric sub-population (patients aged 60 years or younger at study inclusion), as illustrated in **Figure 1**.

### HCMV serostatus is not different between patients with severe COVID-19 and pre-pandemic patients

95 patients suffering from severe COVID-19 with virus induced sepsis and 80 pre-pandemic, post-surgery individuals without signs of infection (controls) were included in this prospective, observational study. 61% of the COVID-19 patients were male and the median age was 58 years (IQR 49-74years). This was not significantly different to the control cohort in which 38% were male (p=0,112) and the median age was 65 (IQR: 57-76) years (p=0,076).

**Figure 2:**
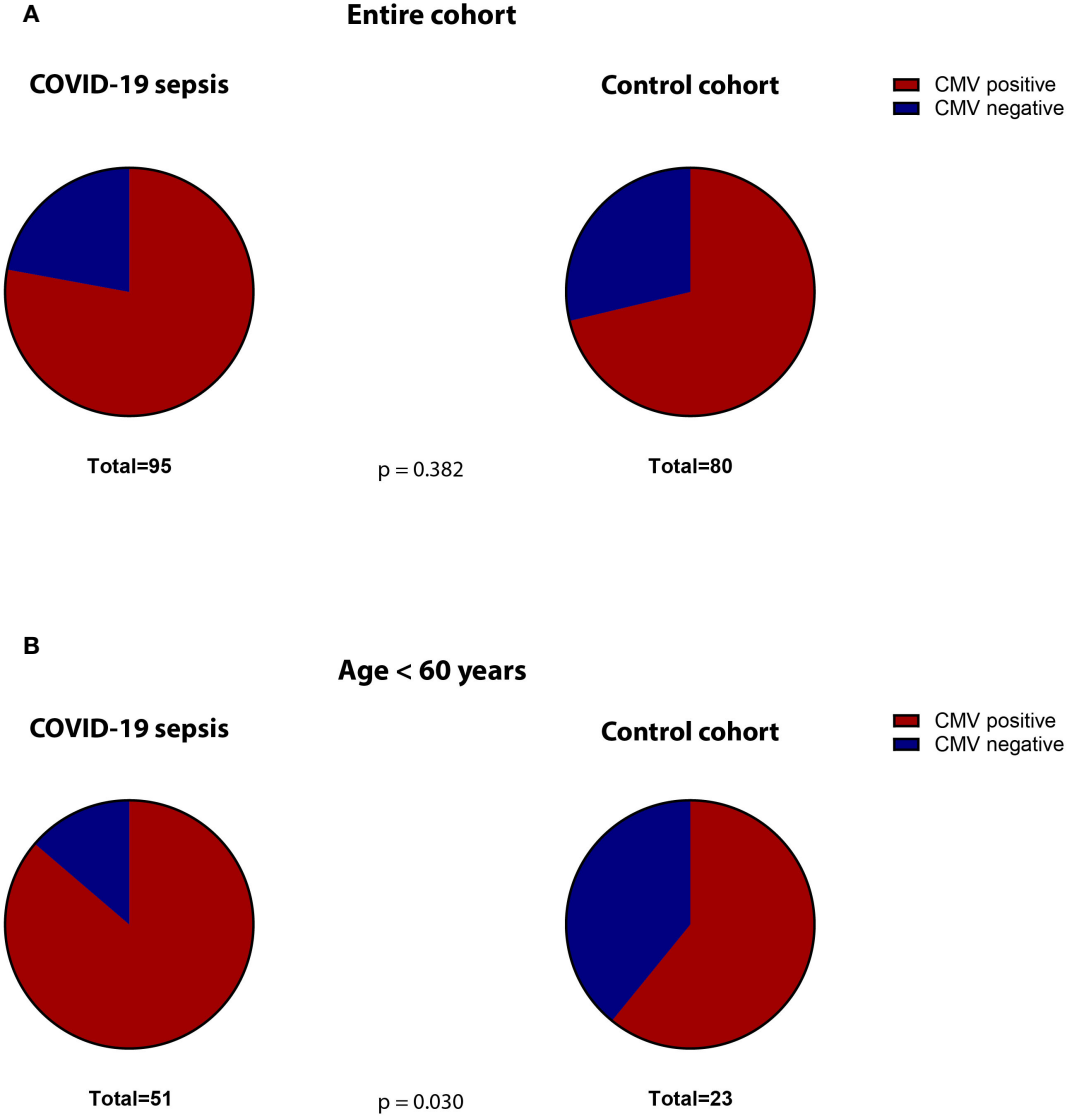
Proportion of CMV-seropositive (red) versus -seronegative (blue) patients in the **(A)** total cohorts (COVID-19 versus controls) and **(B)** in the subgroup of non-geriatric patients (COVID-19 versus controls).

The median SOFA-score at study inclusion was 9 (IQR 5-12) for the COVID-19 cohort. The 30-day mortality was 42%. Co-morbidities were assessed when available. Comparing the frequency of relevant co-morbidities between the COVID-19 and post-surgical patients, we find diabetes (22% vs. 12% respectively, p=0.023) and obesity (37% vs. 21%, p=0.001) to be more frequent in COVID-19 patients. Malignant diseases (5% vs. 80% p=0.001), alcohol abuse (1% vs. 11%, p=0.017) and nicotine addiction (6% vs. 36% p=0.001) were more frequent in controls. 78% of the COVID-19 patients were seropositive for HCMV-IgG at study inclusion. This was not significantly different than the control cohort (71%, p = 0.382, **Figure 2A**).

Additional baseline characteristics are presented in **Table 1** and immune phenotyping with regard to HCMV-serostatus is shown in **Supplementary Table 1**.

**Table 1:** Baseline characteristics of COVID-19 ICU patients and post-surgery control patients.

	COVID-19 induced sepsis n=95	post-surgery patients; n=80	p-value
**gender male**	58 (61%)	35 (48%);	0.117
**CMV-IgG positive at day one**	74 (78%)	57 (71%);	0.382
**age in years median (IQR)**	58 (49-74)	65 (57-76);	0.076
**SOFA score at day one median (IQR)**	9 (5-12)	n.a.	n.a.
Median Oxygenation-Index (paO_2_/FiO_2_) day 1 (median ± IQR)	157 (115-191) n=49	n.a.	n.a.
Mean arterial blood pressure (MAP) day 1 (median ± IQR)	84 (79-92) n=53	n.a.	n.a.
Number of patients receiving adrenalin on day 1	1	n.a.	n.a.
Number of patients receiving dobutamin on day 1	3	n.a.	n.a.
Number of patients receiving noradrenalin on day 1	27	n.a.	n.a.
Platelet count day 1 (lowest value, median ± IQR)	217 (130-283) n=58	n.a.	n.a.
Serum creatinin (mg/dl) day 1 (highest value, median ± IQR)	0,54 (0,34-0,78) n=58	n.a.	n.a.
Serum bilirubine (mg/dl) day 1 (highest value, median ± IQR)	0,88 (0,73-1,33) n= 56	n.a.	n.a.
Co-Morbidities			
- pulmonal (non copd)	10 (11%)	11 (15%)	0.818
- copd	5 (5%)	12 (16%)	0.076
- nicotin	6 (6%)	26 (36%)	0.001
- diabetes	21 (22%)	9 (12%)	0.023
- hypertension	42 (44%)	46 (63%)	0.502
- obesity	35 (37%)	15 (21%)	0.001
- cardiovascular	16 (17%)	23 (32%)	0.195
- malignant	5 (5%)	58 (80%)	0.001
- alcohol	1 (1%)	8 (11%)	0.017
- transplantation	7 (7%)	1 (1%)	0.063
- Kidney (non rrt)	11 (12%)	4 (6%)	0.100
- renal replacement therapy (rrt)	0 (0%)	0 (0%)	n.a.
**length of stay on ICU days median (IQR)**	18 (6-29,5)	n.a.	n.a.
**length of stay in hospital median (IQR)**	21 (12-31,5)	11 (6-21)	0.001
**30-day survival**	55 (57,9%)	80 (100%)	0.001
Leukocyte count (cells*1000/µl) median (IQR) day 1	10,3 (7,2-13,7)	8,5 (6,3-12,5)	0.225

n.a., not applicable.

### In patients 60 years or younger, the frequency of HCMV seropositivity is significantly higher than in comparable control patients

The non-geriatric sub-population of these cohorts (i.e. patients under or equal 60 years of age) consisted of 51 patients with severe COVID-19 and 23 post-surgery individuals. In this cohort, 65% of the COVID-19 patients were male, compared to only 39% in the control cohort (p=0.047). The median age in COVID-19 patients and controls was almost equal (49 [IQR:45-56] vs. 50 [IQR: 35-57] years respectively, p = 0.935). The 30-day survival in these COVID-19 patients was 59%. The median SOFA score at study inclusion was 9 (IQR: 5.5 – 12). When comparing co-morbidities, we find significantly more malignant diseases (5% vs. 87%, p=0.001) as well as a higher frequency of nicotine-abuse in post-surgery patients (10% vs. 30%, p=0.043), which aligns with the general cohort. At the time of admission, 86% of the non-geriatric COVID-19 patients presented sero-positive for HCMV-IgG, while only 61% of the post-surgery controls did so (p=0,030, **Figure 2B**).

**Table 2** depicts patients characteristics of the non-geriatric patients.

**Table 2:** Baseline characteristics of COVID-19 ICU patients and post-surgery control patients aged 18-60years.

	COVID-19 induced sepsis <60years; n=51	postoperative patients <60years; n=23	p-value
**gender male**	33 (65%)	9 (39%)	0.047
**CMV-IgG positive at day one**	44 (86%)	14 (61%)	0.030
**age in years median (IQR)**	49 (45-56)	50 (35-57)	0.935
**SOFA score at day one median (IQR)**	9 (5,5-12)	n.a.	
Co-Morbidities			
- pulmonal (non copd)	3 (7%)	3 (13%)	0.657
- copd	2 (5%)	4 (17%)	0.174
- nicotin	4 (10%)	7 (30%)	0.043
- diabetes	11 (26%)	3 (13%)	0.345
- hypertension	18 (43%)	9 (39%)	0.799
- obesity	24 (57%)	6 (26%)	0.021
- cardiovascular	4 (10%)	2 (8%)	1.000
- malignant	2 (5%)	20 (87%)	0.001
- alcohol	1 (2%)	3 (13%)	0.123
- transplantation	3 (7%)	1 (4%)	1.000
- Kidney (non rrt)	5 (12%)	1 (4%)	0.411
- renal replacement therapy	0 (0%)	0 (0%)	n.a.
**length of stay on ICU days median (IQR)**	21 (6-35)	n.a.	n.a.
**length of stay in hospital median (IQR)**	23 (10,5-42,5)	13 (6-22)	0.025
**30-day survival**	30 (59%)	23 (100%)	0.001

n.a., not applicable.

### HCMV serostatus does not affect 30-day mortality

**Figure 3:**
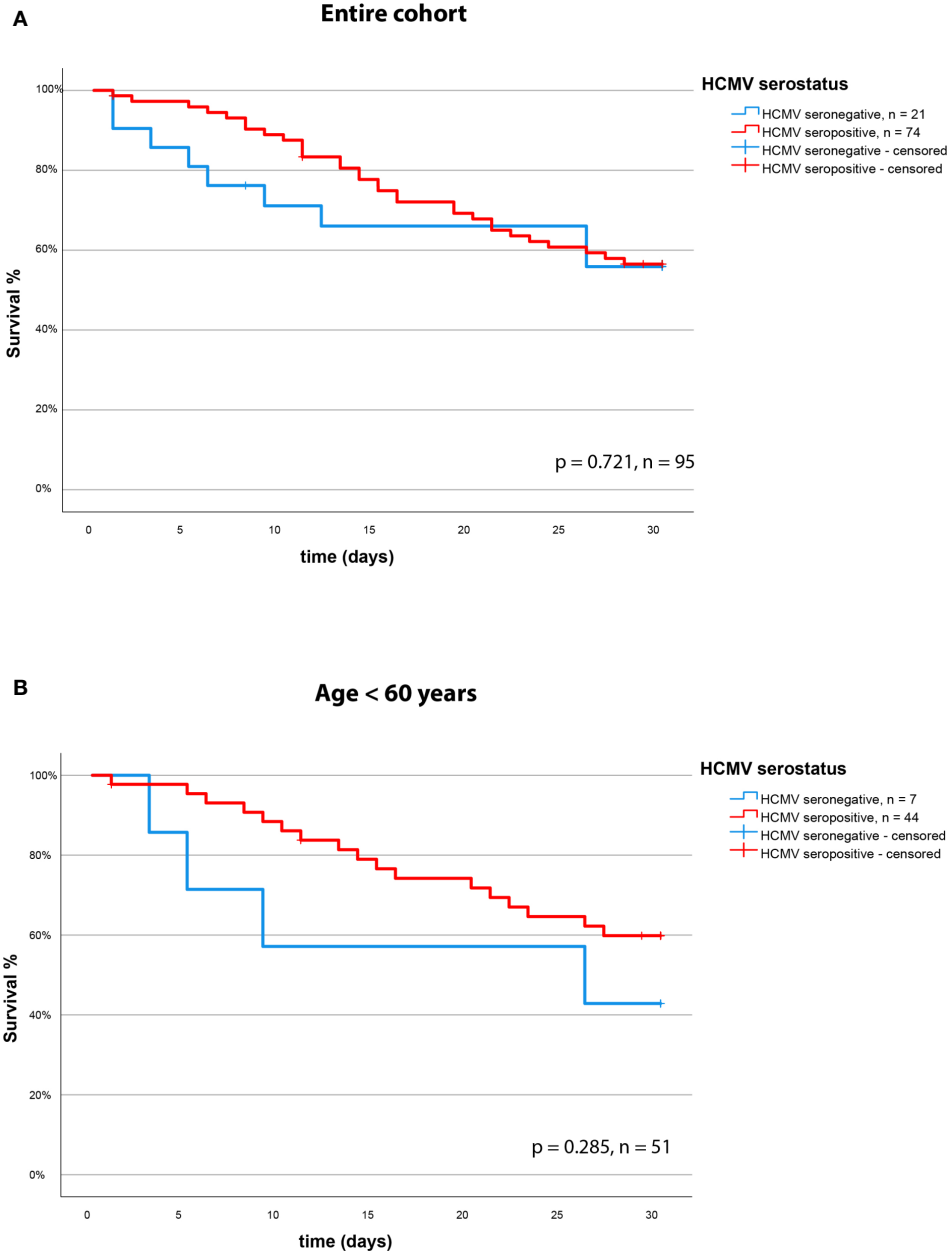
30-day survival (Kaplan-Meier curve) based on HCMV serostatus in **(A)** the overall cohort and **(B)** in the subgroup of non-geriatric patients.

We assessed the effect of the HCMV serostatus on 30-day mortality in the entire cohort of patients with severe COVID-19. We could not identify HCMV seropositivity as a prognostic factor in this cohort. The Kaplan Meier Analysis (**Figures 3A, B**) shows no significant effect (survival: 57% vs. 58% for HCMV seronegative vs. seropositive respectively, p = 0.721, log rank test).

## Discussion

In our study, we observed no significant impact of HCMV serostatus on the 30-day mortality in COVID-19 sepsis. This finding contrasts starkly with bacterial sepsis, where HCMV serostatus was an independent risk factor for mortality (18). The intriguing divergence prompts contemplation on the nuanced interplay between HCMV, T-cell dynamics, and the heterogeneous landscape of COVID-19 sepsis.

The association of HCMV seropositivity with a specialized T-cell pool and diminished naïve T-cell reservoirs, known as T-cell inflation (36), has been postulated to render HCMV-seropositive patients more susceptible to heterologous infections, as their T-cell repertoire is significantly diminished (37). This might well be an explanation for the effects of HCMV on bacterial sepsis patients (18).

However, why does this not translate to COVID-19 sepsis? Interestingly, HCMV specific CD8^+^ T-cells have been shown to react to SARS-CoV-2 peptides (38) just as SARS-CoV-2 T-cell reacted to HCMV (33). This would grant HCMV seropositive patients a certain protection from COVID-19 induced death, as T-cell function is an important factor when it comes to SARS-CoV-2 immunity (39).

But how does this fit to the findings of others, that have reported that HCMV might be a risk factor for severe COVID-19 in non-geriatric patients (34)? This is especially interesting as we find the same effect of COVID-19 patients under the age of 60 more frequently being HCMV seropositive than post-surgical control patients of the same age group.

We assume, the explanation lies in the characteristic timeline of T-cell inflation, which unfolds over a longer lifetime due to recurrent HCMV reactivations (37). Consequently, any discernible effects on the immune system may be more pronounced in older patients, thereby explaining the association of HCMV with COVID-19 sepsis in younger adults.

Crucially, our study’s design deviates from previous works, such as Weber et al. (34), by contrasting severe COVID-19 cases with a pre-pandemic cohort of post-surgery patients devoid of infection or sepsis development. This approach enhances the generalizability of our findings beyond specific COVID-19 patient subsets, thereby augmenting the external validity of our results. This becomes particularly crucial when considering potential interventions or preventive measures based on our observations. What needs to be discussed at this point, is the main and obvious condition, distinguishing our post-surgery cohort from the COVID-19 patients under the age of 60 years: One main reason for surgery were malignant diseases. Thus, we cannot exclude that the known association between HCMV and malignancies (40) (41) plays further role. In this light the association does not contradict our interpretation, as we find even higher rates of HCMV-seropositivity in the COVID-19 patients than in the post-surgery cohort.

Future investigations should delve into the intricate interactions among HCMV, the immune system, and the pathogenesis of COVID-19-induced sepsis, with a specific focus on delineating the role of T-cell function and its implications for disease outcomes. By unraveling the underlying mechanisms, exploring associations in diverse patient populations, and scrutinizing potential interventions, we can deepen our understanding of HCMV’s impact on COVID-19 and potentially enhance patient management strategies.

Nevertheless, our study harbors limitations that warrant acknowledgment. Despite a relatively larger COVID-19 cohort compared to previous studies, the sample size remains modest, particularly when undertaking subgroup analyses. Thus, we advocate for retrospective assessments of HCMV serostatus in larger observational COVID-19 trials to validate our findings rigorously. The prevalence of HCMV seropositivity in the non-geriatric COVID-19 cohort introduces another constraint, diminishing the size of the seronegative cohort. As such, caution is warranted, and we refrain from definitively ruling out an effect of HCMV serostatus on the 30-day mortality in the non-geriatric cohort, given our limited statistical power.

In conclusion, our investigation unveils that HCMV seropositivity does not exert a discernible effect on the 30-day mortality in COVID-19 patients. However, a nuanced association surfaces, suggesting HCMV as a potential risk factor for severe disease, particularly in younger patients. This dichotomy underscores the complexity of viral-bacterial interactions within the immune landscape and underscores the need for further extensive studies to refine our comprehension.

## Data availability statement

The raw data supporting the conclusions of this article will be made available by the authors, without undue reservation.

## Ethics statement

The studies involving humans were approved by Ethics Committee of the Medical Faculty of Ruhr-University Bochum. The studies were conducted in accordance with the local legislation and institutional requirements. The participants provided their written informed consent to participate in this study.

## Author contributions

DZ: Investigation, Writing – original draft, Writing – review & editing. AWo: Conceptualization, Data curation, Validation, Writing – original draft, Writing – review & editing. TR: Project administration, Writing – review & editing. HN: Software, Validation, Writing – review & editing. HH: Data curation, Writing – original draft. LB: Resources, Writing – review & editing. KR: Methodology, Resources, Writing – original draft. BD: Investigation, Writing – review & editing. LP: Data curation, Investigation, Writing – original draft. BM: Data curation, Writing – original draft. AWi: Data curation, Writing – review & editing. KW: Data curation, Writing – review & editing. SP: Supervision, Writing – review & editing. ME: Conceptualization, Investigation, Writing – review & editing. MoA: Conceptualization, Writing – review & editing. NB: Conceptualization, Writing – review & editing. TB: Conceptualization, Data curation, Methodology, Writing – review & editing. BS: Conceptualization, Methodology, Writing – review & editing. MB: Data curation, Writing – original draft. AZ: Conceptualization, Data curation, Writing – review & editing. TV: Data curation, Writing – review & editing. CP: Conceptualization, Writing – review & editing. SE: Data curation, Formal Analysis, Writing – original draft. CW: Data curation, Writing – review & editing. MiA: Conceptualization, Data curation, Funding acquisition, Project administration, Resources, Supervision, Writing – review & editing. MU: Data curation, Investigation, Methodology, Resources, Visualization, Writing – original draft. BK: Conceptualization, Formal Analysis, Funding acquisition, Investigation, Methodology, Project administration, Supervision, Writing – original draft, Writing – review & editing.

## Supplementary material

Supplementary Figure 1
**(A)** Proportion of CMV-seropositive (red) versus -seronegative (blue) patients in the geriatric (>=60years) cohort (COVID-19 and controls). **(B)** 30-day survival (Kaplan-Meier curve) based on HCMV serostatus in the geriatric COVID-19 cohort (>=60years).
