# Supplementary figures and images for: Exploring the relationship between HCMV serostatus and outcomes in COVID-19 sepsis
Source: Front Immunol. 2024 May 8;15:1386586. doi: 10.3389/fimmu.2024.1386586 (PMC11109369; doi:10.3389/fimmu.2024.1386586)

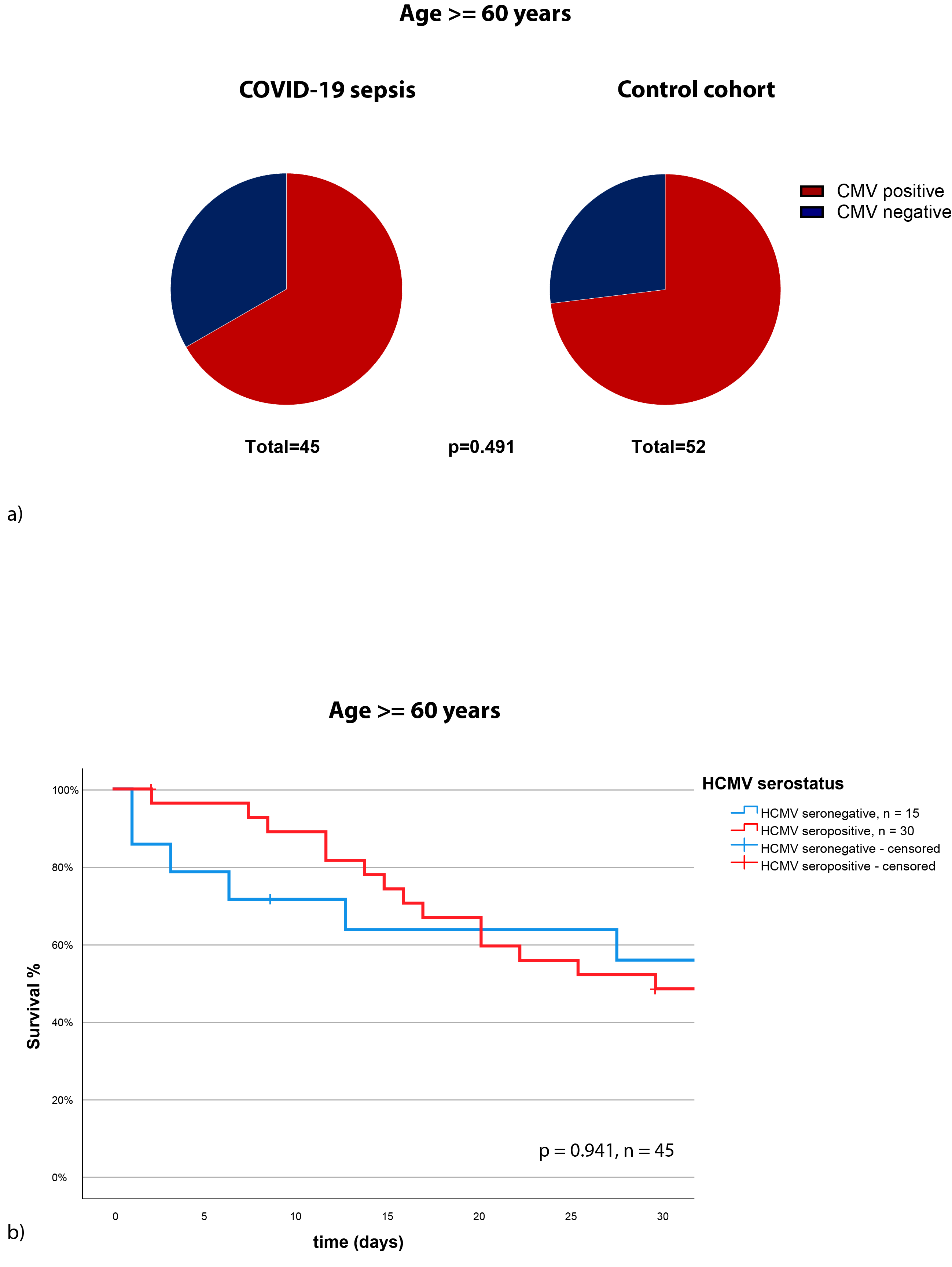

Supplement: Supplementary Figure 1 — (A) Proportion of CMV-seropositive (red) versus -seronegative (blue) patients in the geriatric (>=60years) cohort (COVID-19 and controls). (B) 30-day survival (Kaplan-Meier curve) based on HCMV serostatus in the geriatric COVID-19 cohort (>=60years). [file Image_1.png]
