# Supplementary material for: Exploring the relationship between HCMV serostatus and outcomes in COVID-19 sepsis
Source: Front Immunol. 2024 May 8;15:1386586. doi: 10.3389/fimmu.2024.1386586 (PMC11109369; doi:10.3389/fimmu.2024.1386586)
Supplement: Supplementary file 2 [file Table_1.docx]

| **Zellen** | **Gesamt** | **pos** | **neg** | **p** | **n** |
| --- | --- | --- | --- | --- | --- |
| Lympho to Monocyte Ratio | 0,66 | 0,66 | 0,69 | 0,605 | 77 |
| Lymphocytes in PBMCs (%) | 14 | 15 | 12 | 0,249 | 77 |
| Monozytes in PBMCs (%) | 8 | 9 | 5 | 0,103 | 77 |
| B-Cells in Lymphocytes (%) | 19 | 18 | 23 | 0,342 | 77 |
| NK cells in Lymphocytes (%) | 17 | 15 | 20 | 0,204 | 77 |
| NKT cells in Lymphocytes (%) | 6 | 6 | 6 | 0,867 | 77 |
| T-cells in Lymphocytes (%) | 46 | 48 | 40 | 0,103 | 77 |
| CD4 T-cells in Lymphocytes (%) | 25 | 26 | 23 | 0,675 | 77 |
| Memory CD4 T-cells in Lymphocytes (%) | 16 | 16 | 14 | 0,396 | 77 |
| Central Memory CD4 T-cells in Lymphocytes (%) | 8 | 8 | 8 | 0,915 | 77 |
| Effector Memory CD4 T-cells in Lymphocytes (%) | 7 | 7 | 5 | 0,078 | 77 |
| Naive CD4 T-cells in Lymphocytes (%) | 10 | 10 | 9 | 0,868 | 77 |
| TEMRA CD4 T-cells in Lymphocytes (%) | 0,2 | 0,2 | 0,2 | 0,945 | 77 |
| CD8 T-cells in Lymphocytes (%) | 13 | 15 | 9 | 0,021 | 77 |
| Memory CD8 T-cells in Lymphocytes (%) | 11 | 12 | 7 | 0,05 | 77 |
| Central Memory CD8 T-cells  in Lymphocytes (%) | 1 | 1,1 | 1 | 0,827 | 77 |
| Effector Memory CD8 T-cells  in Lymphocytes (%) | 5 | 6 | 3 | 0,185 | 77 |
| Naive CD8 T-cells in Lymphocytes (%) | 3 | 3 | 2 | 0,255 | 77 |
| TEMRA CD8 T-cells in Lymphocytes (%) | 4 | 5 | 3 | 0,05 | 77 |
| Monocytes (%) | 15 | 16 | 11 | 0,204 | 77 |
| Classical monocytes in  monocytes (%) | 85 | 87 | 78 | 0,084 | 77 |
| Intermediate monocytes in monocytes (%) | 9 | 7 | 16 | 0,001 | 77 |
| Non-classical monocytes in monocytes (%) | 5 | 5 | 6 | 0,639 | 77 |

Supplemental Table 1: Immune-cell composition in COVID-19 sepsis patients.
